# Clinical Spectrum and Exploratory Mortality Markers in Hospitalized Patients with Different Clinical Presentations of West Nile Virus Infection: A Multicenter Cohort from Southern Italy

**DOI:** 10.3390/medicina62071364

**Published:** 2026-07-15

**Authors:** Pierpaolo Di Micco, Giuseppe Cardillo, Egidio Imbalzano, Rodolfo Nasti, Maria Gabriella Coppola, Concetta Schiano, Carmine Siniscalchi

**Affiliations:** 1 Internal Medicine Ward, P.O. Santa Maria delle Grazie, 80078 Naples, Italy; 2 Laboratory Unit, MedyLab Srl., 40128 Bologna, Italy; giuseppe.cardillo75@gmail.com; 3 Department of Internal Medicine, Messina University Hospital, 98121 Messina, Italy; egidio.imbalzano@unime.it; 4 Internal Medicine Unit, Ospedale Evangelico Villa Betania, 80147 Naples, Italy; rodolfo.nasti@gmail.com; 5 Internal Medicine Ward, Benevento Hospital, 82100 Benevento, Italy; gabry.cop@libero.it; 6 Department of Advanced Medical and Surgical Sciences (DAMSS), University of Campania Luigi Vanvitelli, 80138 Naples, Italy; concetta.schiano@unicampania.it; 7 Internal Medicine Department, Parma University Hospital, 43125 Parma, Italy; csiniscalchi84@gmail.com

**Keywords:** West Nile virus, West Nile fever, neuroinvasive disease, atypical presentation, oligosymptomatic infection, clinical spectrum, disease progression, internal medicine, viral infection, lymphocytopenia

## Abstract

*Background and Objectives*: West Nile virus (WNV) infection has a broad clinical spectrum ranging from asymptomatic or mild disease to severe neuroinvasive forms. Data on hospitalized patients with atypical or non-neurological presentations remain limited. *Materials and Methods*: We conducted a retrospective multicenter observational study including 30 patients with laboratory-confirmed WNV infection admitted to internal medicine wards during a seasonal outbreak in Southern Italy. Clinical characteristics, hematological parameters, and in-hospital outcomes were analyzed. *Results*: Median age was 77 years, and 53% of patients were male. Neurological involvement was observed in 50% of patients, whereas 37% presented with septic-like manifestations. Overall in-hospital mortality was 43%, and early mortality within 10 days occurred in 17%. Neurological involvement was significantly more frequent among non-survivors and was associated with increased in-hospital mortality. Higher monocyte percentages were associated with overall mortality, whereas eosinopenia, leukopenia, and thrombocytopenia were associated with early mortality in exploratory analyses. *Conclusions*: Hospitalized patients with WNV infection may present with heterogeneous clinical phenotypes extending beyond classical neuroinvasive disease. Neurological involvement appears to be the main clinical marker of adverse outcome, while selected hematological parameters may provide preliminary prognostic information. Given the limited sample size and retrospective design, these findings should be considered hypothesis-generating.

## 1. Introduction

West Nile virus (WNV) infection is an emerging mosquito-borne disease caused by an RNA flavivirus belonging to the *Flaviviridae* family. Since its introduction into Europe, WNV has progressively become a relevant public health concern, particularly in Mediterranean countries [1] characterized by favorable ecological conditions for viral transmission [2]. Seasonal outbreaks are increasingly reported in Southern Europe, including Italy, where recurrent epidemic waves have been documented over the last decade [3,4,5,6].

The clinical spectrum of WNV infection is remarkably heterogeneous. Approximately 80% of infected individuals remain asymptomatic, whereas nearly 20% develop a self-limited febrile illness characterized by fatigue, fever, headache, myalgia, arthralgia, or gastrointestinal symptoms. Less than 1% of infected patients develop neuroinvasive disease, including meningitis, encephalitis, or acute flaccid paralysis [7,8], which represents the most severe manifestation of the infection [9,10].

Most available studies on WNV have focused on neuroinvasive disease because of its substantial morbidity and mortality, while few data are available for patients affected by WNV without neurological involvement. However, this traditional dichotomous classification between mild infection and neuroinvasive disease may not fully reflect the complexity of real-world clinical presentations observed in hospitalized patients because of the possible presence of chronic comorbidity that may influence the clinical outcome. In internal medicine settings, WNV infection may present with atypical or nonspecific manifestations, including septic-like syndromes, isolated inflammatory abnormalities, mild constitutional symptoms, or subtle neurological changes that may initially escape recognition [5].

In recent years, increasing evidence has highlighted the role of systemic inflammation and host response in determining disease severity across several infectious and inflammatory conditions. Routine hematological parameters and composite inflammatory indices derived from peripheral blood counts have emerged as potentially useful potential prognostic markers in acute diseases, including viral infections and thromboembolic disorders [11,12].

Similarly, during the COVID-19 pandemic, several studies demonstrated associations between immune dysregulation, hematological abnormalities, frailty, and adverse clinical outcomes [13,14,15,16]. These observations support the hypothesis that host-related inflammatory and immunological factors may contribute substantially to the clinical heterogeneity observed in WNV infection.

Nevertheless, data regarding laboratory exploratory markers of mortality and disease progression in hospitalized patients with WNV infection remain limited, especially outside dedicated neurological cohorts. Furthermore, little is known about the evolution of patients initially presenting with oligosymptomatic or atypical forms of disease.

The aim of the present study was therefore to describe the real-world clinical spectrum of WNV infection in hospitalized patients admitted to internal medicine wards during a localized outbreak in Southern Italy and to explore clinical and hematological variables associated with short-term mortality and in-hospital outcomes. Given the limited sample size, the present analyses were considered exploratory and hypothesis-generating.

## 2. Material and Methods

### 2.1. Study Design and Population

We conducted a retrospective multicenter observational cohort study including consecutive adult patients admitted to internal medicine wards of four hospitals in Southern Italy during a localized seasonal outbreak of West Nile virus (WNV) infection.

Medical records of patients admitted for fever of unknown origin, suspected infectious disease, or neurological symptoms potentially attributable to viral infection were systematically reviewed. Eligible patients were identified at the time of hospital admission and followed throughout their hospitalization.

*Inclusion criteria were:*

1. Laboratory-confirmed WNV infection, defined by serological positivity for anti-WNV IgM and IgG antibodies and/or molecular confirmation by polymerase chain reaction (PCR);
2. Availability of clinical and laboratory data at hospital admission.

Patients with incomplete baseline clinical data or uncertain microbiological diagnosis were excluded from the analysis.

The study was conducted according to the principles of the Declaration of Helsinki. Given the retrospective observational design and the use of anonymized clinical data, formal ethics committee approval was not required according to local institutional regulations.

### 2.2. Clinical Phenotypes

Patients were retrospectively classified according to their clinical presentation at hospital admission into three predefined phenotypic groups:

• **Neurologic phenotype**

Defined by the presence of neurological manifestations suggestive of neuroinvasive disease, including altered mental status, encephalopathy, headache, meningismus, nausea and vomiting associated with neurological symptoms, axial rigidity, confusion, or focal neurological signs.

• **Septic-like phenotype**

Defined by systemic inflammatory manifestations mimicking bacterial sepsis, including fever, tachypnea, respiratory symptoms, hypotension, or urinary symptoms, in the absence of predominant neurological involvement at presentation.

• **Oligosymptomatic/atypical phenotype**

Defined by mild or nonspecific symptoms such as asthenia, low-grade fever, generalized malaise, or minimal systemic complaints without clear neurologic or septic features.

Phenotype adjudication was performed retrospectively through comprehensive review of clinical records, admission notes, laboratory findings, imaging studies, and clinical evolution during hospitalization. Classification was independently performed by two clinicians at each participating center, and disagreements were resolved by consensus discussion.

Sepsis was defined according to Sepsis-3 criteria as suspected or documented infection associated with an acute increase of at least two points in the Sequential Organ Failure Assessment (SOFA) score. Neuroinvasive disease was defined according to established clinical and laboratory criteria, including neurological manifestations associated with cerebrospinal fluid abnormalities and/or compatible neuroimaging findings.

### 2.3. Data Collection

Clinical and laboratory data were collected from electronic medical records using standardized extraction procedures.

Baseline variables included:

• Demographic characteristics;
• Comorbidities;
• Presenting symptoms;
• Neurological involvement;
• Septic features;
• Previous antithrombotic therapies;
• Hematological and biochemical parameters at admission.

Laboratory variables included complete blood count with leukocyte differential, platelet count, coagulation parameters, and routine biochemical tests.

The following hematological variables were specifically analyzed:

• Leukocyte count;
• Neutrophil percentage;
• Lymphocyte percentage;
• Monocyte percentage;
• Eosinophil percentage;
• Basophil percentage;
• Hemoglobin;
• Hematocrit;
• Platelet count.

### 2.4. Clinical Outcomes and Evolution

Two main outcomes were evaluated:

1. Early mortality. Defined as death occurring within 10 days from hospital admission for any reason after WNV infection.
2. Overall in-hospital mortality. Defined as death occurring during hospitalization or within 30 days from admission for any reason after WNV infection.

Cross-tabulation of mortality variables demonstrated complete overlap between cumulative 30-day mortality and in-hospital mortality. Therefore, these variables were unified into a single overall mortality endpoint.

Secondary outcomes included:

• Progression from oligosymptomatic to neurologic or septic-like phenotype;
• Progression from septic-like to neurologic phenotype;
• Persistence of the initial clinical phenotype;
• Length of hospital stay;
• Intensive care unit admission.

### 2.5. Laboratory Analysis

For the purpose of the present analysis, only laboratory parameters measured at hospital admission were considered. Although repeated measurements were available during hospitalization, no longitudinal or time-dependent analyses were performed due to the limited sample size and heterogeneity in sampling intervals.

Hematological parameters were measured using automated analyzers routinely employed at each participating center, according to local standard operating procedures.

### 2.6. Statistical Analysis

Given the exploratory nature of the study and the limited sample size, no formal sample size calculation was performed before study initiation. All consecutive eligible patients admitted during the outbreak period were included.

Continuous variables are reported as median and interquartile range (IQR), whereas categorical variables are expressed as counts and percentages.

Comparisons between survivors and non-survivors were performed using:

• Mann–Whitney U test for continuous variables;
• Fisher’s exact test for categorical variables.

Receiver operating characteristic (ROC) curve analyses were performed to evaluate the discriminatory performance of continuous hematological variables for mortality outcomes. Areas under the curve (AUCs) with 95% confidence intervals were calculated using bootstrap resampling (1000 repetitions). ROC-derived exploratory cut-off values were identified using the Youden index.

Univariate logistic regression analyses were performed to explore associations between clinical variables and mortality outcomes. Odds ratios (ORs) with 95% confidence intervals (CIs) were reported. In contingency tables with zero-cell frequencies, Haldane–Anscombe correction was applied.

Because of the limited number of events (5 deaths at 10 days and 13 overall deaths), multivariable regression models were not considered reliable according to the events-per-variable rule. Consequently, all regression analyses were restricted to univariate exploratory models, and findings should be interpreted as hypothesis-generating.

Variables with more than 50% missing values were excluded a priori from statistical analyses. No correction for multiple comparisons was applied because of the exploratory design of the study. Consequently, statistically significant findings should be interpreted with caution, as the large number of comparisons increases the possibility of false-positive associations. All statistical analyses were performed using Python version 3.11 (pandas, SciPy, statsmodels, and scikit-learn libraries). A two-sided *p*-value < 0.05 was considered statistically significant.

## 3. Results

### 3.1. Baseline Characteristics of the Cohort

A total of 30 patients with laboratory-confirmed West Nile virus infection were included in the study. Median age was 77 years (IQR 66.5–81.0), and 16 patients (53.3%) were male. Neurological manifestations at presentation were observed in 15 patients (50.0%), whereas 11 patients (36.7%) presented with a septic-like clinical phenotype. Baseline characteristics of the overall cohort are summarized in Table 1.

The study population was characterized by substantial comorbidity burden. Heart failure was present in 63.3% of patients, chronic obstructive pulmonary disease (COPD) in 23.3%, active or previous cancer in 20.7%, and chronic dialysis treatment in 6.7%. Antithrombotic therapy before admission was reported in nearly half of the patients.

Hematological evaluation at admission demonstrated frequent inflammatory and immunological abnormalities. Median leukocyte count was 7.70 × 10^9^/L (IQR 6.40–10.20), whereas median lymphocyte percentage was 11.0% (IQR 8.0–13.6). Median platelet count was 180 × 10^9^/L (IQR 130–224).

### 3.2. Mortality Outcomes

Early mortality within 10 days occurred in 5 patients (16.7%), whereas overall in-hospital mortality occurred in 13 patients (43.3%).

Cross-tabulation of mortality variables demonstrated that cumulative 30-day mortality completely overlapped with in-hospital mortality. Therefore, these variables were analyzed as a unified endpoint representing total mortality.

### 3.3. Factors Associated with Early Mortality

Comparisons between survivors and patients who died within 10 days are summarized in Table 2.

Patients who experienced early mortality showed significantly lower leukocyte counts at admission compared with survivors (5.50 vs. 7.85 × 10^9^/L; *p* = 0.037). Eosinophil counts were also markedly lower among non-survivors, with eosinopenia strongly associated with early mortality (*p* = 0.007).

Chronic dialysis treatment was more frequent among patients who died within 10 days (40% vs. 0%; *p* = 0.023). Similarly, abnormal activated partial thromboplastin time (aPTT) values were more commonly observed in early non-survivors (40% vs. 0%; *p* = 0.028). Thrombocytopenia also showed a strong association with early mortality, although the number of events was limited.

No significant associations were observed for age, sex, COPD, heart failure, cancer, or previous antithrombotic therapies.

### 3.4. Factors Associated with Overall In-Hospital Mortality

Comparisons between survivors and patients who died during hospitalization are reported in Table 3.

Neurological involvement was significantly more frequent among patients who died compared with survivors (77% vs. 29%; *p* = 0.025). Monocyte percentages were also significantly higher in non-survivors (7.1% vs. 3.9%; *p* = 0.018). When patients were stratified according to the presence or absence of neurological involvement, in-hospital mortality was 66.7% among patients with neurological manifestations (10/15) compared with 20.0% among those without neurological involvement (3/15). This difference was statistically significant (*p* = 0.025), supporting the role of neurological presentation as the main clinical marker of adverse outcome in this cohort.

Although eosinophil counts tended to be lower among patients who died, the difference did not reach statistical significance (*p* = 0.073).

No statistically significant differences were observed for age, leukocyte count, platelet count, hemoglobin levels, COPD, heart failure, cancer, or previous antithrombotic therapy.

Age was not significantly associated with overall in-hospital mortality (75 vs. 79 years; *p* = 0.154).

### 3.5. ROC Curve Analyses

ROC analyses were performed to evaluate the discriminatory performance of baseline hematological variables for mortality outcomes. For 10-day mortality, eosinophil count showed the highest apparent discriminatory performance, with an AUC of 0.96 (95% CI 0.87–1.00). However, given the very limited number of events and the presence of missing eosinophil values, this estimate should be interpreted with considerable caution and regarded as an exploratory finding. The ROC-derived cut-off corresponded to complete eosinopenia, yielding 100% sensitivity and 92% specificity in this dataset. This threshold should be considered exploratory only and should not be interpreted as a clinically applicable cut-off. Leukocyte count also showed good discriminatory performance, with an AUC of 0.80 (95% CI 0.55–0.96).

**Figure 1:**
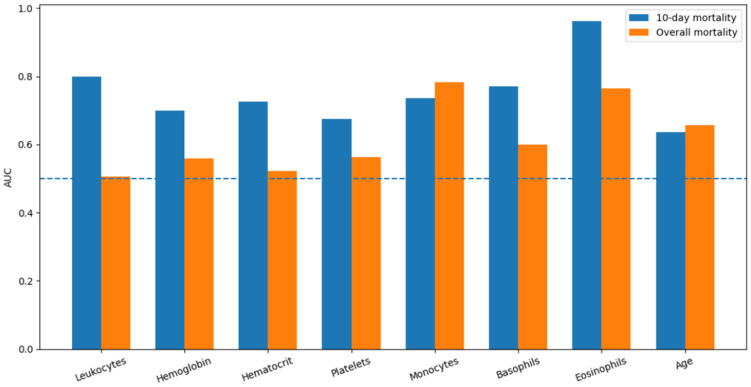
Summary of ROC curve analyses for hematological variables associated with mortality outcomes. Legend: Summary of area under the curve (AUC) values for continuous hematological variables evaluated as exploratory markers of 10-day mortality and overall in-hospital mortality in hospitalized patients with West Nile virus infection. Variables with AUC ≥ 0.75 showed the highest apparent discriminatory performance within this exploratory cohort. Eosinophil count showed the highest predictive accuracy for early mortality, whereas monocyte percentage demonstrated the best performance for overall mortality. The dashed horizontal line represents the reference value for random discrimination (AUC = 0.50). The corresponding ROC-derived exploratory cut-off values for each parameter are reported in Supplementary Tables S1 and S2.

For overall in-hospital mortality, monocyte percentage showed the highest discriminatory performance, with an AUC of 0.78 (95% CI 0.59–0.96). Eosinophil count also showed relatively good discrimination for overall mortality, with an AUC of 0.76. A graphical summary of the main ROC findings is shown in Figure 1. Detailed ROC analyses are reported in Supplementary Tables S1 and S2, while representative ROC curves are provided in Supplementary Figures S1 and S2.

### 3.6. Univariate Logistic Regression Analyses

Exploratory univariate logistic regression analyses were performed to evaluate clinical and laboratory variables associated with mortality outcomes. Thrombocytopenia was significantly associated with mortality within 10 days, whereas lower leukocyte count showed a non-significant trend toward increased early mortality.

For overall in-hospital mortality, neurological involvement emerged as the strongest clinical exploratory marker of death. Patients with neurological manifestations had approximately eight-fold higher odds of in-hospital mortality compared with those without neurological involvement. Higher monocyte percentages were also significantly associated with overall mortality.

Given the exploratory nature of the study and the limited number of events, these findings should be interpreted with caution. Detailed univariate logistic regression models are reported in Supplementary Tables S3 and S4.

## 4. Discussion

In the present multicenter observational study, we evaluated the clinical spectrum and exploratory mortality markers of hospitalized patients with West Nile virus infection admitted to internal medicine wards during a localized outbreak in Southern Italy. Our findings confirm the marked heterogeneity of WNV clinical presentation and suggest that selected clinical and hematological parameters may provide preliminary hypothesis-generating information in hospitalized patients.

A first relevant observation emerging from our cohort is the high frequency of non-classical presentations. Although neuroinvasive disease remains the most recognized manifestation of severe WNV infection, a substantial proportion of patients in our cohort initially presented with septic-like or oligosymptomatic phenotypes. These findings reinforce the concept that WNV infection should not be viewed exclusively as a neurological disease but rather as a systemic viral condition characterized by heterogeneous inflammatory and immunological responses. This aspect is particularly relevant in internal medicine settings, where patients frequently present with nonspecific symptoms and multiple comorbidities that may complicate early diagnosis [4,5,6,7,8,9].

The overall mortality observed in our cohort was relatively high, with 43% of patients dying during hospitalization. While for inpatients with encephalitis the increased mortality rate is expected per se, for inpatients with different clinical presentation the increased mortality rate seems to be related to the complexity of clinical scenario. This finding, in fact, likely reflects the advanced age and clinical complexity of hospitalized patients rather than the intrinsic lethality of WNV infection itself. Indeed, our study population included frail elderly individuals with a high prevalence of cardiovascular disease, chronic comorbidities, and neurological involvement. In this context, recent evidence has also emphasized that severe WNV infection may be associated with cardiovascular involvement, including myocardial injury, myocarditis, arrhythmias, and hemodynamic instability, particularly in patients with severe systemic or neuroinvasive disease. This aspect may be clinically relevant in older hospitalized patients with pre-existing cardiovascular comorbidities and may contribute to adverse outcomes [9]. Previous studies have consistently shown that advanced age and neuroinvasive disease represent the main determinants of poor outcomes in WNV infection [10,17].

One of the most robust findings of the present study was the association between neurological involvement and overall mortality. Patients presenting with neurological manifestations showed an approximately eight-fold increased risk of in-hospital death. This observation is coherent with the established literature regarding neuroinvasive WNV disease, which is associated with substantial mortality and long-term neurological sequelae [9,10]. Neurological manifestations likely reflect both direct viral neurotropism [18] and severe systemic inflammatory activation [19]. Furthermore, neurological impairment may contribute indirectly to adverse outcomes through respiratory complications, aspiration risk, prolonged immobilization, and increased susceptibility to secondary infections.

Besides the lymphocytopenia commonly observed in viral infections, including WNV infection, other hematological parameters may also have prognostic value and contribute to the heterogeneous clinical manifestations of the disease [20]. In our cohort, higher monocyte percentages were associated with increased overall mortality and showed relatively good discriminatory performance in ROC analyses. Monocytes play a central role in the innate immune response [21], cytokine activation during viral infections [22], endothelial dysfunction [23], and neuroinflammation with subsequent tissue damage in severe viral diseases [11,12,13,14,15,24]. Although monocyte-related indices have been extensively investigated in thromboembolic and inflammatory conditions, including acute pulmonary embolism [25], evidence in WNV infection remains limited. Therefore, our findings identify a potentially relevant area for future investigation.

Another interesting observation concerns eosinophil depletion. Complete eosinopenia demonstrated remarkably high discriminatory performance for early mortality, with an AUC approaching 1.0. However, this apparently excellent discriminatory performance should be interpreted with extreme caution. Given the small number of mortality events, the presence of missing eosinophil data, and the exploratory nature of the analyses, the observed AUC may reflect overfitting or sampling variability rather than true predictive performance. Similar findings have been described in bacterial sepsis and severe viral infections, including COVID-19, where eosinopenia has been interpreted as a marker of acute systemic stress and immune dysregulation. However, caution is required in interpreting this result because eosinophil values were unavailable in a substantial proportion of patients.

Lower leukocyte counts were also associated with early mortality. Leukopenia and lymphocyte alterations are well-recognized laboratory features of WNV infection and may reflect direct viral effects on immune regulation or bone marrow response. Similar associations between leukocyte abnormalities and adverse outcomes have been reported in other systemic viral infections [13,14,15].

Thrombocytopenia emerged as another variable [26] associated with early mortality. Although the confidence intervals were extremely wide because of the small number of events, the direction of the association is biologically plausible. Platelet consumption and coagulation abnormalities frequently occur during severe inflammatory and infectious conditions and may reflect systemic endothelial activation and disease severity [14].

The present study has several important limitations. First, the sample size was very small, with only 30 patients and a limited number of mortality events. Consequently, statistical power was low, confidence intervals were wide, and multivariable regression analyses were not considered reliable according to standard events-per-variable recommendations. All associations should therefore be interpreted as exploratory and hypothesis-generating.

Second, several laboratory variables, particularly eosinophils and basophils, showed substantial missing data, potentially introducing selection bias and limiting the robustness of the ROC analyses. In addition, because numerous hematological variables were explored without correction for multiple comparisons, some statistically significant findings may represent chance associations.

Fourth, the retrospective design inherently limits causal inference and may introduce information bias. Important markers of disease severity, including SOFA score, Glasgow Coma Scale, frailty indices, and longitudinal laboratory trends, were not consistently available. Furthermore, no survival analysis could be performed because exact time-to-event data were unavailable.

An additional limitation is that WNV testing was not systematically performed in all hospitalized patients during the outbreak period. Consequently, undiagnosed WNV infections may have been missed, potentially introducing selection bias. Furthermore, only patients admitted to Internal Medicine wards were included in this study. Patients managed exclusively in Neurology or those admitted directly to the Intensive Care Unit without subsequent transfer to Internal Medicine were not represented. Therefore, the present cohort may not fully reflect the entire spectrum of hospitalized WNV infection, particularly the most severe neurological or critical care presentations.

Despite these limitations, the study also presents several strengths. We analyzed a real-world multicenter cohort of hospitalized patients managed in internal medicine settings rather than highly selected neurological units. This approach allowed us to capture the broader and more heterogeneous clinical spectrum of WNV infection, including atypical and oligosymptomatic forms that are frequently underrepresented in the literature. Given the small sample size, the present results should not be interpreted as definitive prognostic models. Rather, they provide preliminary clinical signals that may help generate hypotheses for larger prospective studies focused on risk stratification in hospitalized patients with WNV infection.

Overall, our findings support the concept that WNV infection is a dynamic and heterogeneous systemic disease extending beyond classical neuroinvasive presentations. Neurological involvement appears strongly associated with mortality, while selected hematological parameters may provide additional prognostic information. Larger prospective studies are needed to validate these exploratory observations and to better define clinically useful risk stratification models in hospitalized patients with WNV infection.

## 5. Conclusions

West Nile virus infection in hospitalized patients is characterized by a broad and heterogeneous clinical spectrum extending beyond classical neuroinvasive disease. In our multicenter cohort, neurological involvement was strongly associated with increased in-hospital mortality and appeared to be the main clinical marker of severe disease.

Selected hematological parameters, including higher monocyte percentage, eosinopenia, leukopenia, and thrombocytopenia, showed exploratory associations with adverse outcomes and may reflect systemic inflammatory and immune dysregulation during infection.

Given the small sample size, missing data, and retrospective design, these findings should be interpreted with caution and considered hypothesis-generating. Larger prospective studies are required to validate clinically useful risk stratification models in hospitalized patients with WNV infection.

## Figures and Tables

**Table 1:** Baseline characteristics of the overall study population.

Variable	Overall Cohort (*N* = 30)
Demographic characteristics	
Age, years	77.0 [66.5–81.0]
Male sex, *n* (%)	16 (53.3)
Clinical characteristics and comorbidities	
Neurological involvement, *n* (%)	15 (50.0)
Septic-like phenotype, *n* (%)	11 (36.7)
COPD, *n* (%)	7 (23.3)
Heart failure, *n* (%)	19 (63.3)
Dialysis, *n* (%)	2 (6.7)
Thrombocytopenia, *n* (%)	3 (10.0)
Satellite lymphangitis, *n* (%)	4 (13.8)
Cancer, *n* (%)	6 (20.7)
PT within normal range, *n*/*N* (%)	23/27 (85.2)
aPTT within normal range, *n*/*N* (%)	25/27 (92.6)
Antithrombotic therapy, *n*/*N* (%)	12/26 (46.2)
Antiplatelet therapy, *n*/*N* (%)	12/26 (46.2)
Anticoagulant therapy, *n*/*N* (%)	6/26 (23.1)
Hematological parameters	
Red blood cells, ×10^12^/L	3.87 [3.65–4.36]
Leukocytes, ×10^9^/L	7.70 [6.40–10.20]
Hemoglobin, g/dL	11.40 [10.30–13.00]
Hematocrit, %	37.60 [32.60–40.20]
MCV, fL	83.50 [77.10–89.00]
MCH, pg	29.00 [28.00–30.30]
MCHC, g/dL	32.50 [31.03–33.80]
Platelet count, ×10^9^/L	180 [130–224]
Neutrophils, %	78.70 [77.00–81.50]
Lymphocytes, %	11.00 [8.00–13.60]
Monocytes, %	4.50 [3.00–7.60]
Basophils, %	0.07 [0.00–0.12]
Eosinophils, %	0.10 [0.00–0.30]

Legend: Continuous variables are reported as median and interquartile range (IQR). Categorical variables are reported as absolute counts and percentages. COPD: chronic obstructive pulmonary disease; PT: prothrombin time; aPTT: activated partial thromboplastin time; MCV: mean corpuscular volume; MCH: mean corpuscular hemoglobin; MCHC: mean corpuscular hemoglobin concentration.

**Table 2:** Comparison between survivors and non-survivors at 10 days.

Variable	Survivors (*n* = 25)	Non-Survivors (*n* = 5)	*p*-Value
Demographic characteristics			
Age, years	78.0 [68.0–82.0]	72.0 [64.0–79.0]	0.358
Male sex, *n* (%)	13 (52)	3 (60)	1.000
Clinical characteristics and comorbidities			
Neurological involvement, *n* (%)	11 (44)	4 (80)	0.330
Septic-like phenotype, *n* (%)	10 (40)	1 (20)	0.626
COPD, *n* (%)	7 (28)	0 (0)	0.304
Heart failure, *n* (%)	15 (60)	4 (80)	0.626
Dialysis, n (%)	0 (0)	2 (40)	**0.023**
Thrombocytopenia, *n* (%)	1 (4)	2 (40)	0.064
PT within normal range, *n*/*N* (%)	19/22 (86)	4/5 (80)	1.000
aPTT within normal range, *n*/*N* (%)	22/22 (100)	3/5 (60)	**0.028**
Antithrombotic therapy, *n*/*N* (%)	10/21 (48)	2/5 (40)	1.000
Antiplatelet therapy, *n*/*N* (%)	11/21 (52)	1/5 (20)	0.330
Anticoagulant therapy, *n*/*N* (%)	4/21 (19)	2/5 (40)	0.558
Satellite lymphangitis, *n* (%)	3/24 (12)	1/5 (20)	0.553
Cancer, *n* (%)	5/24 (21)	1/5 (20)	1.000
Hematological parameters			
Red blood cells, ×10^12^/L	4.04 [3.73–4.40]	3.83 [3.65–3.96]	0.377
Leukocytes, ×10^9^/L	7.85 [6.85–10.49]	5.50 [4.39–6.40]	**0.037**
Hemoglobin, g/dL	11.65 [10.65–13.20]	10.30 [9.80–11.90]	0.185
Hematocrit, %	38.20 [34.38–40.50]	32.60 [31.00–35.00]	0.134
MCV, fL	82.00 [77.07–89.53]	85.10 [85.00–88.50]	0.519
MCH, pg	29.00 [27.75–30.07]	30.10 [28.00–31.10]	0.290
MCHC, g/dL	32.10 [31.01–33.65]	33.79 [33.23–34.00]	0.262
Platelet count, ×10^9^/L	180 [137.50–225.25]	165 [98.00–182.00]	0.236
Neutrophils, %	78.85 [76.58–82.25]	78.00 [77.00–79.20]	0.496
Lymphocytes, %	11.00 [7.00–14.20]	11.00 [11.00–11.90]	0.563
Monocytes, %	4.00 [2.75–7.60]	7.10 [4.50–11.00]	0.117
Basophils, %	0.10 [0.01–0.20]	0.00 [0.00–0.03]	0.116
Eosinophils, %	0.10 [0.10–0.30]	0.00 [0.00–0.00]	**0.007**

Legend: Continuous variables are reported as median and interquartile range (IQR) and were compared using the Mann–Whitney U test. Categorical variables are expressed as absolute counts and percentages and were compared using Fisher’s exact test. COPD: chronic obstructive pulmonary disease; PT: prothrombin time; aPTT: activated partial thromboplastin time; MCV: mean corpuscular volume; MCH: mean corpuscular hemoglobin; MCHC: mean corpuscular hemoglobin concentration. Statistically significant *p*-values are reported in bold.

**Table 3:** Comparison between survivors and non-survivors for overall in-hospital mortality.

Variable	Survivors (*n* = 17)	Non-Survivors (*n* = 13)	*p*-Value
Demographic characteristics			
Age, years	79.0 [68.0–84.0]	75.0 [64.0–79.0]	0.154
Male sex, *n* (%)	8 (47)	8 (62)	0.484
Clinical characteristics and comorbidities			
Neurological involvement, *n* (%)	5 (29)	10 (77)	**0.025**
Septic-like phenotype, *n* (%)	8 (47)	3 (23)	0.259
COPD, *n* (%)	4 (24)	3 (23)	1.000
Heart failure, *n* (%)	11 (65)	8 (62)	1.000
Dialysis, *n* (%)	0 (0)	2 (15)	0.179
Thrombocytopenia, *n* (%)	1 (6)	2 (15)	0.565
PT within normal range, *n*/*N* (%)	12/14 (86)	11/13 (85)	1.000
aPTT within normal range, *n*/*N* (%)	14/14 (100)	11/13 (85)	0.222
Antithrombotic therapy, *n*/*N* (%)	5/14 (36)	7/12 (58)	0.431
Antiplatelet therapy, *n*/*N* (%)	6/14 (43)	6/12 (50)	1.000
Anticoagulant therapy, *n*/*N* (%)	3/14 (21)	3/12 (25)	1.000
Satellite lymphangitis, *n* (%)	1/16 (6)	3/13 (23)	0.299
Cancer, *n* (%)	4/16 (25)	2/13 (15)	0.663
Hematological parameters			
Red blood cells, ×10^12^/L	4.05 [3.62–4.40]	3.83 [3.65–4.25]	0.765
Leukocytes, ×10^9^/L	7.37 [6.65–10.21]	7.80 [5.50–9.60]	0.983
Hemoglobin, g/dL	11.65 [10.50–13.00]	11.00 [10.30–13.00]	0.643
Hematocrit, %	38.20 [33.28–39.42]	35.00 [32.60–40.20]	0.870
MCV, fL	79.00 [76.75–89.53]	84.00 [82.00–88.50]	0.355
MCH, pg	28.50 [26.38–30.12]	30.00 [28.00–30.30]	0.172
MCHC, g/dL	32.09 [30.50–33.84]	32.50 [31.43–33.79]	0.724
Platelet count, ×10^9^/L	180 [140.50–228.50]	165 [124.00–201.00]	0.583
Neutrophils, %	79.20 [76.55–83.25]	78.70 [77.00–80.40]	0.723
Lymphocytes, %	10.00 [6.95–16.50]	11.00 [10.00–12.40]	0.785
Monocytes, %	3.90 [2.00–4.50]	7.10 [4.50–10.00]	**0.018**
Basophils, %	0.07 [0.02–0.18]	0.05 [0.00–0.10]	0.536
Eosinophils, %	0.30 [0.08–0.30]	0.05 [0.00–0.10]	0.073

Legend: Continuous variables are reported as median and interquartile range (IQR) and were compared using the Mann–Whitney U test. Categorical variables are expressed as absolute counts and percentages and were compared using Fisher’s exact test. COPD: chronic obstructive pulmonary disease; PT: prothrombin time; aPTT: activated partial thromboplastin time; MCV: mean corpuscular volume; MCH: mean corpuscular hemoglobin; MCHC: mean corpuscular hemoglobin concentration. Statistically significant *p*-values are reported in bold.

## Data Availability

All data generated or analysed in this study are included in this published article. Additional details may be provided by the corresponding author upon reasonable request.

## Supplementary Materials

The following supporting information can be downloaded at https://www.mdpi.com/article/10.3390/medicina62071364/s1. Figure S1: ROC curves of hematological variables associated with 10-day mortality; Figure S2: ROC curves of hematological variables associated with overall in-hospital mortality; Table S1: Receiver operating characteristic (ROC) analysis for 10-day mortality; Table S2: Receiver operating characteristic (ROC) analysis for overall in-hospital mortality; Table S3: Univariate logistic regression analyses for 10-day mortality; Table S4: Univariate logistic regression analyses for overall in-hospital mortality.
