# Supplementary material for: Clinical Spectrum and Exploratory Mortality Markers in Hospitalized Patients with Different Clinical Presentations of West Nile Virus Infection: A Multicenter Cohort from Southern Italy
Source: Medicina (Kaunas). 2026 Jul 15;62(7):1364. doi: 10.3390/medicina62071364 (PMC13414162; doi:10.3390/medicina62071364)
Supplement: Supplementary file 1 [file medicina-62-01364-s001.zip › medicina-4393801-supplementary.pdf]

Supplementary Table S1. Receiver operating characteristic (ROC) analysis for 10-day mortality.

Variable	AUC (95% CI)	Direction	ROC-derived exploratory cut-off	Sensitivity	Specificity
Age	0.636 (0.331–0.901)	↓	81.0	100%	28%
Red blood cells	0.635 (0.382–0.879)	↓	4.25	100%	40%
Leukocytes	0.800 (0.551–0.962)	↓	≤6.4 $\times 10^9$/L	80%	83%
Hemoglobin	0.700 (0.445–0.917)	↓	11.9 g/dL	100%	45%
Hematocrit	0.725 (0.423–0.957)	↓	35.0%	80%	60%
MCV	0.600 (0.257–0.864)	↑	85.0 fL	80%	70%
MCH	0.660 (0.326–0.957)	↑	31.1 pg	40%	95%
MCHC	0.670 (0.258–0.964)	↑	33.23 g/dL	80%	70%
Platelet count	0.675 (0.393–0.923)	↓	193 $\times 10^9$/L	100%	42%
Neutrophils	0.605 (0.348–0.833)	↓	80.8%	100%	35%
Lymphocytes	0.590 (0.333–0.849)	↑	11.0%	100%	50%
Monocytes	0.735 (0.480–0.945)	↑	4.3%	100%	55%
Basophils	0.771 (0.450–0.962)	↓	0.0%	75%	75%
Eosinophils	0.962 (0.875–1.000)	↓	0.0%	100%	92%

Legend: ROC analyses were performed to evaluate the discriminatory performance of continuous hematological variables for early mortality within 10 days. AUC: area under the curve; CI: confidence interval; Sens: sensitivity; Spec: specificity. ROC-derived exploratory cut-off values were identified using the Youden index. Direction indicates whether lower (↓) or higher (↑) values were associated with mortality.

Supplementary Table S2. Receiver operating characteristic (ROC) analysis for overall in-hospital mortality.

Variable	AUC (95% CI)	Direction	ROC-derived exploratory cut-off	Sensitivity	Specificity
Age	0.656 (0.457–0.844)	↓	82.0	100%	35%
Red blood cells	0.538 (0.288–0.783)	↓	4.20	69%	50%
Leukocytes	0.505 (0.270–0.737)	↑	7.70 $\times 10^9$/L	62%	56%
Hemoglobin	0.558 (0.317–0.793)	↓	11.0 g/dL	54%	67%
Hematocrit	0.522 (0.277–0.763)	↓	37.6%	62%	58%
MCV	0.612 (0.360–0.824)	↑	81.0 fL	85%	58%
MCH	0.663 (0.441–0.873)	↑	28.0 pg	100%	42%
MCHC	0.545 (0.313–0.773)	↑	31.43 g/dL	85%	50%
Platelet count	0.562 (0.347–0.776)	↓	229 $\times 10^9$/L	92%	25%

https://doi.org/10.3390/xxxxx

Variable	AUC (95% CI)	Direction	ROC-derived exploratory cut-off	Sensitivity	Specificity
Neutrophils	0.545 (0.299–0.792)	↓	80.8%	85%	42%
Lymphocytes	0.535 (0.276–0.805)	↑	8.0%	92%	42%
Monocytes	0.782 (0.587–0.958)	↑	≥4.3%	85%	75%
Basophils	0.600 (0.300–0.864)	↓	0.0%	50%	83%
Eosinophils	0.764 (0.493–0.967)	↓	0.0%	50%	100%

Legend: ROC analyses were performed to evaluate the discriminatory performance of continuous hematological variables for overall in-hospital mortality. AUC: area under the curve; CI: confidence interval; Sens: sensitivity; Spec: specificity. ROC-derived exploratory cut-off values were identified using the Youden index. Direction indicates whether lower (↓) or higher (↑) values were associated with mortality.

Supplementary Table S3. Univariate logistic regression analyses for 10-day mortality.

Variable	OR (95% CI)	p-value	N analyzed	Events
Age	0.95 (0.88–1.03)	0.234	30	5
Red blood cells	0.44 (0.07–2.99)	0.405	25	5
Leukocytes	0.65 (0.40–1.05)	0.077	29	5
Hemoglobin	0.60 (0.28–1.28)	0.188	25	5
Hematocrit	0.81 (0.63–1.05)	0.118	25	5
MCV	1.02 (0.93–1.12)	0.629	25	5
MCH	1.19 (0.78–1.80)	0.413	25	5
MCHC	1.09 (0.76–1.58)	0.644	25	5
Platelet count	0.99 (0.97–1.01)	0.289	29	5
Neutrophils	0.98 (0.86–1.13)	0.813	25	5
Lymphocytes	1.02 (0.90–1.15)	0.767	25	5
Monocytes	1.25 (0.93–1.69)	0.140	25	5
Basophils	Non-estimable	0.195	16	4
Eosinophils	—	—	—	—
Male sex	1.38 (0.20–9.77)	0.744	30	5
Neurological involvement	5.09 (0.50–52.29)	0.171	30	5
Septic-like phenotype	0.38 (0.04–3.87)	0.410	30	5
COPD	—	—	—	—
Heart failure	2.67 (0.26–27.49)	0.410	30	5
Dialysis	—	—	—	—
Thrombocytopenia	16.00 (1.09–234.26)	**0.043**	30	5

Variable	OR (95% CI)	p-value	N analyzed	Events
PT within normal range	0.63 (0.05–7.75)	0.719	27	5
aPTT within normal range	—	—	—	—
Antithrombotic therapy	0.73 (0.10–5.33)	0.759	26	5
Antiplatelet therapy	0.23 (0.02–2.39)	0.217	26	5
Anticoagulant therapy	2.83 (0.35–23.01)	0.330	26	5
Satellite lymphangitis	1.75 (0.14–21.39)	0.661	29	5
Cancer	0.95 (0.09–10.50)	0.967	29	5

Legend: Odds ratios (ORs) with 95% confidence intervals (CIs) were calculated using univariate logistic regression models. Variables marked as "non-estimable" indicate perfect or near-perfect separation due to the limited number of events. COPD: chronic obstructive pulmonary disease; PT: prothrombin time; aPTT: activated partial thromboplastin time. Statistically significant p-values are reported in bold.

Supplementary Table S4. Univariate logistic regression analyses for overall in-hospital mortality.

Variable	OR (95% CI)	p-value	N analyzed	Events
Age	0.95 (0.89–1.02)	0.175	30	13
Red blood cells	1.01 (0.23–4.56)	0.986	25	13
Leukocytes	1.00 (0.79–1.28)	0.976	29	13
Hemoglobin	0.91 (0.54–1.52)	0.720	25	13
Hematocrit	0.96 (0.81–1.15)	0.675	25	13
MCV	1.03 (0.96–1.10)	0.389	25	13
MCH	1.23 (0.85–1.77)	0.275	25	13
MCHC	1.02 (0.78–1.33)	0.894	25	13
Platelet count	1.00 (0.99–1.00)	0.653	29	13
Neutrophils	1.03 (0.91–1.16)	0.682	25	13
Lymphocytes	0.98 (0.88–1.09)	0.688	25	13
Monocytes	1.36 (1.01–1.83)	**0.043**	25	13
Basophils	Non-estimable	0.808	16	10
Eosinophils	0.03 (0.00–6.35)	0.203	17	10
Male sex	1.80 (0.41–7.81)	0.433	30	13

Variable	OR (95% CI)	p-value	N analyzed	Events
Neurological involvement	8.00 (1.52–42.04)	**0.014**	30	13
Septic-like phenotype	0.34 (0.07–1.68)	0.184	30	13
COPD	0.97 (0.18–5.39)	0.977	30	13
Heart failure	0.87 (0.20–3.90)	0.858	30	13
Dialysis	—	—	—	—
Thrombocytopenia	2.91 (0.23–36.17)	0.406	30	13
PT within normal range	0.92 (0.11–7.67)	0.936	27	13
aPTT within normal range	Non-estimable	1.000	27	13
Antithrombotic therapy	2.52 (0.52–12.30)	0.253	26	12
Antiplatelet therapy	1.33 (0.28–6.28)	0.716	26	12
Anticoagulant therapy	1.22 (0.20–7.59)	0.830	26	12
Satellite lymphangitis	4.50 (0.41–49.63)	0.219	29	13
Cancer	0.55 (0.08–3.59)	0.528	29	13

Legend: Odds ratios (ORs) with 95% confidence intervals (CIs) were calculated using univariate logistic regression models. Variables marked as "non-estimable" indicate perfect or near-perfect separation due to the limited number of events. COPD: chronic obstructive pulmonary disease; PT: prothrombin time; aPTT: activated partial thromboplastin time. Statistically significant p-values are reported in bold.

Supplementary Figure S1. ROC curves of hematological variables associated with 10-day mortality

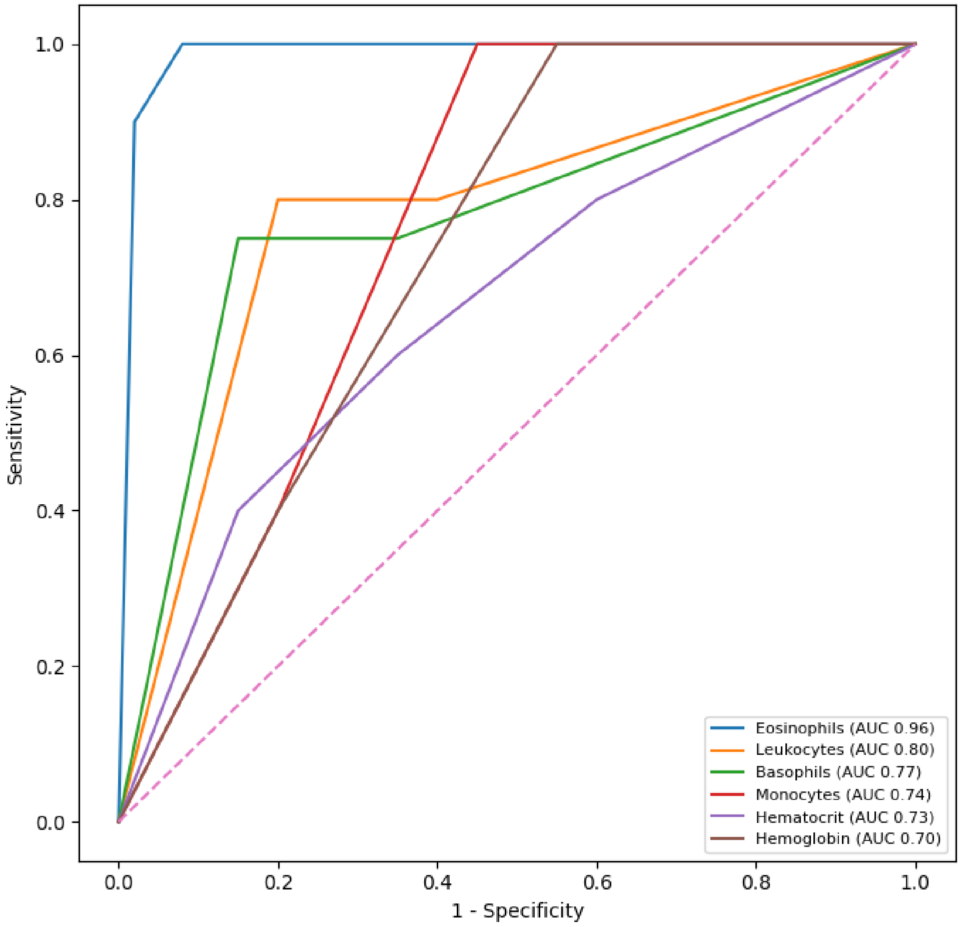

Legend: Receiver operating characteristic (ROC) curves for the hematological variables showing the highest discriminatory performance for early mortality within 10 days. Eosinophil count showed the highest apparent discriminatory performance (AUC 0.96), although this finding should be interpreted cautiously because of the limited sample size and missing values, followed by leukocyte count (AUC 0.80), basophils (AUC 0.77), and monocytes (AUC 0.74). Lower eosinophil and leukocyte values were associated with increased mortality risk. The corresponding ROC-derived exploratory cut-off values identified by the Youden index are reported in Supplementary Table S1.

https://doi.org/10.3390/xxxxx

Supplementary Figure S2. ROC curves of hematological variables associated with overall in-hospital mortality

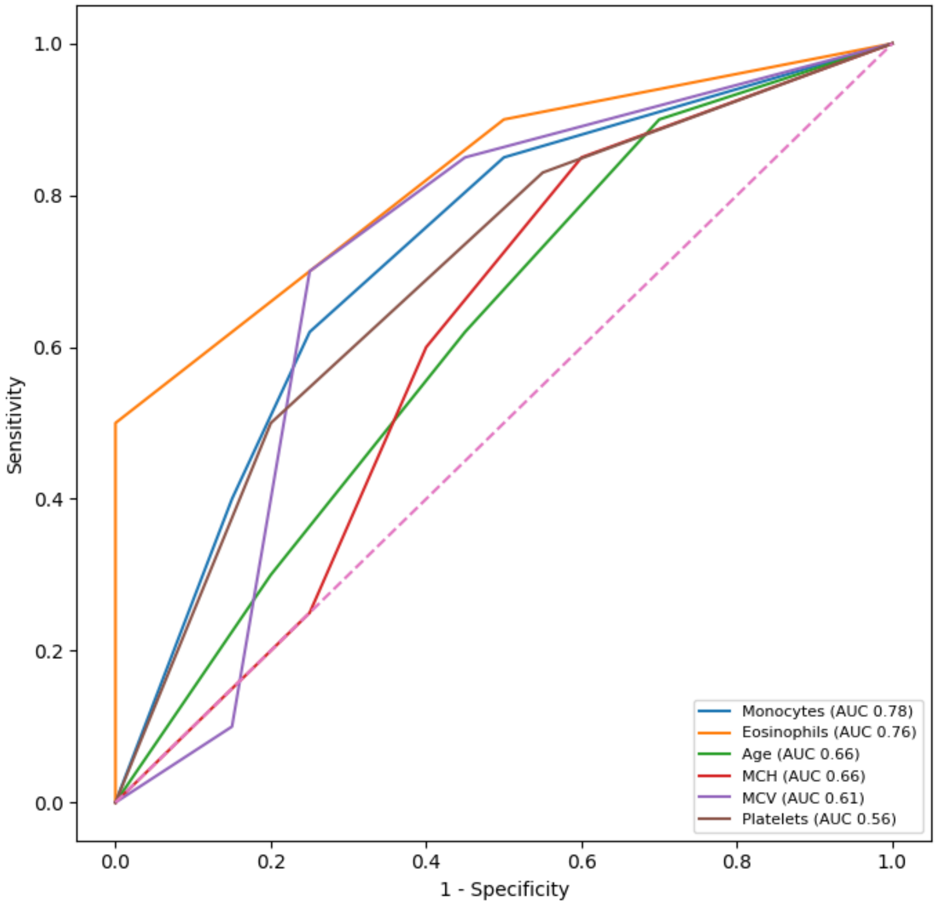

Legend: Receiver operating characteristic (ROC) curves for the hematological variables with the highest discriminatory performance for overall in-hospital mortality. Monocyte percentage demonstrated the best apparent discriminatory performance (AUC 0.78), followed by eosinophil count (AUC 0.76). Higher monocyte values and lower eosinophil values were associated with increased mortality risk. The corresponding ROC-derived exploratory cut-off values identified by the Youden index are reported in Supplementary Table S2.

https://doi.org/10.3390/xxxxx
